# Correlations between IgG4-related disease, autoimmune pancreatitis, and allergic diseases

**DOI:** 10.3389/fimmu.2026.1718303

**Published:** 2026-05-19

**Authors:** Corina Porr, Anca Vidrighin, Dana M. Harris, Cosmina Diaconu

**Affiliations:** 1 Faculty of Medicine, Lucian Blaga University, Sibiu, Romania; 2 Internal Medicine Department, Mayo Clinic, Jacksonville, FL, United States

**Keywords:** allergy, IgG4, IgG4-related disease, lymphoplasmacytic infiltrate, type 1 autoimmune pancreatitis

## Abstract

IgG4-related disease (IgG4-RD) is a relatively recently described condition whose etiology and pathophysiology remain unclear. Histopathological features include dense lymphoplasmacytic tissue infiltration with numerous IgG4-positive plasma cells, storiform fibrosis, and obliterative phlebitis. Most individuals with IgG4-RD are asymptomatic; however, the disease can affect nearly any organ, leading to a wide range of clinical presentations. The four most common patterns are hepatopancreatobiliary disease, systemic disease, head and neck disease, and retroperitoneal disease. Diagnosis is challenging due to diverse organ involvement and is most often confirmed by biopsy. Elevated serum IgG4 concentration can support the diagnosis, though high IgG4 levels are neither highly sensitive nor specific for IgG4-RD. Immunohistochemical IgG4 staining is crucial, especially in patients without elevated serum IgG4. Differential diagnosis is necessary, as IgG4+ plasma cell infiltration is also seen in inflammatory and malignant conditions. First-line treatment consists of glucocorticoids (prednisone), although favorable outcomes have also been achieved with rituximab. Prognosis varies: the disease may resolve spontaneously or follow a relapsing–remitting course. If left untreated or undertreated, IgG4-RD can cause irreversible organ damage and even death. Finally, correlations have been made between IgG4 and different allergic reactions.

## Introduction

IgG is the most common form of immunoglobulins. It accounts for 75% of circulating antibodies, which are an essential part of the secondary immune response to infections and toxins. IgG is produced by plasma cells, a specific type of B lymphocytes.

There are four subclasses of IgG, one of these being IgG4, accounting for approximately 4% of serum IgG, but in chronic allergies, its relative count is elevated by up to 80%. IgG4 has a unique structure and a poor capacity for activating complement and forming immune complexes. Its biological role is not well-known; however, it plays a role in protection against type 1 hypersensitivity reactions (e.g., to bee venom) and in the development of tolerance in atopic patients. It has been shown that beekeepers develop an IgG4-based response to bee venom associated with the reduction of symptoms after exposure to the toxin, and the same event has also been described in other cases of occupational exposure (1–3). IgG4 also plays a role in the pathogenesis of some autoimmune diseases (e.g., pemphigus vulgaris), helminthic infestations, or malignancy (e.g., melanoma), probably due to its anti-inflammatory role (1).

IgG4-related disease (IgG4-RD) is a relatively newly described rare syndrome (4–6), characterized by fibro-inflammatory histopathology in multiple organs with a tendency to form tumefactive lesions and often elevated serum IgG4 concentrations. The histopathology is characterized by a dense lymphoplasmacytic infiltration of tissues with many IgG4-positive plasma cells, storiform fibrosis, and obliterative phlebitis. Elevated serum IgG4 levels are present in only 60%–70% of patients (7–9). The affected organs are the pancreas, the biliary tree, salivary glands, periorbital tissues, kidneys, lungs, lymph nodes, meninges, aorta, breast, skin, prostate, thyroid, and pericardium (7, 8). IgG4-RD is analogous to sarcoidosis, another systemic disease with a unique histological appearance, but disorders like Mikulicz’s syndrome (symmetric lacrimal and salivary gland diseases—dacryoadenitis and sialoadenitis), Küttner’s tumor (chronic sclerosing submandibular sialoadenitis), Riedel’s thyroiditis, Ormond’s disease (retroperitoneal fibrosis), and eosinophilic angiocentric fibrosis are included in IgG4-RD (7, 9, 10).

## Epidemiology

IgG4-RD generally affects people in their sixth to seventh decade of life, but the disease has also been described in children (10, 11). Pancreatobiliary and retroperitoneal localization of the disease demonstrates a male predominance, while head and neck localization most commonly affects women (11).

IgG4-RD most frequently affects the pancreas, but cases have been reported involving nearly every organ (9). Although the reported proportion of organs involved varies widely in the literature, a report found that 60% of cases involved the pancreas, 34% involved the salivary glands, 23% the lacrimal glands, 23% the kidneys, 20% the aorta, 13% the biliary tract, other 13% the lungs, 4% the periorbital tissue, and another 4% involved the retroperitoneum (11). Up to 39% of the patients have a previous or concomitant diagnosis of diabetes mellitus, probably because of the pancreatic involvement (9, 11). The involvement of a single organ is exceptional, IgG4-RD being a multisystem disease in most cases (9, 12).

IgG4, involved in helminthic infestations, also has an indirect effect on allergic disease: patients with chronic parasitic infestation show lower levels of IgE reactivity to dust antigens (13).

Although IgG4-RD is generally considered a rare disease, the number of patients diagnosed with IgG4-RD is increasing, probably because of more research on this condition in the last years.

## Pathogenesis

The etiology of IgG4-RD remains poorly understood. Environmental exposures and genetic factors might place the patients at an increased risk. Some self-antigens (e.g., lactoferrin), microorganisms, allergens, or occupational antigens (e.g., solvents, industrial gases, oil products) may trigger IgG4-RD (8). It may be an autoimmune disease, because antinuclear antibodies and antipancreatic antibodies have been found in autoimmune pancreatitis. It also exhibits features of an allergic disorder, with high Th2 cytokines in tissues, increased IgE levels, and a raised eosinophil count observed in the blood of 40% of patients. Although not specific, most patients with IgG4-RD have an elevated serum IgG4 concentration.

From the viewpoint of IgG4 function, it remains unclear whether IgG4-RD is an autoimmune or an allergic disease. The degree of elevation is correlated with organ involvement and risk of relapse (10, 14, 15). It remains unknown whether IgG4 is directly involved in pathogenesis, is a compensatory response to immune activation, or is simply an epiphenomenon related to a misdirected immune response (10). IgG4 has a poor capacity for activating complement and forming immune complexes. Therefore, it has been postulated that it has an anti-inflammatory role, as it competitively blocks other much more active subtypes (9). However, antigen-specific IgG4 antibodies can be pathogenic in myasthenia gravis, pemphigus foliaceus or vulgaris, thrombotic thrombocytopenia purpura, some forms of membranous glomerulonephritis, and chronic inflammatory demyelinated polyradiculoneuropathy (10).

When a self-antigen or foreign antigen triggers the immune response, the proportion of follicular helper T (Tfh2) cells increases, and B cells are activated. Tfh2 cells produce transforming growth factor-β, which activates fibroblasts that mediate the extracellular matrix remodeling and tissue damage. They also play a key role in the activation of B cells and their differentiation into antibody-producing cells (16). Tfh2 cells also produce interleukins like IL-4, IL-10, and IL-21, involved in the immunoglobulin class switch to IgG4. Several observations support a central role of B cells, because the pathological hallmarks include a lymphoplasmacytic infiltrate rich in IgG4+ plasma cells; because patients have oligoclonal expanded plasma blasts, which decrease in remission; and because the resurgence of plasma blasts and memory B cells is correlated with an increase of disease activity (10, 17, 18). Despite these observations, the precise role of B cells remains uncertain.

Circulating Tfh2 cells are expanded in IgG4-RD. Their frequency is correlated with the number of involved organs, number of plasma blasts, and IgG4 concentration. These cells produce IL-4, which is involved in class switching of B cells to IgG4 and IgE. Dysregulated immunity, characterized by an expansion of CD4^+^ cytotoxic T lymphocytes, seems to be a key hallmark of the disease. These cells are found in the affected tissue, and they decline in number after treatment. They express some mediators of cytotoxicity and produce profibrotic cytokines (10, 19).

The clonal expansion of both plasma blasts and CD4^+^ cytotoxic T lymphocytes seen in patients with IgG4-RD suggests that there might be a common antigen (a viral infection or other unidentified antigen) driving the disease, maybe an autoantigen like annexin A11 and galactin-3 (20). Future work must clarify if T and B cells are responding to the same antigen, and the antigen must be identified (10, 17).

**Figure 1:**
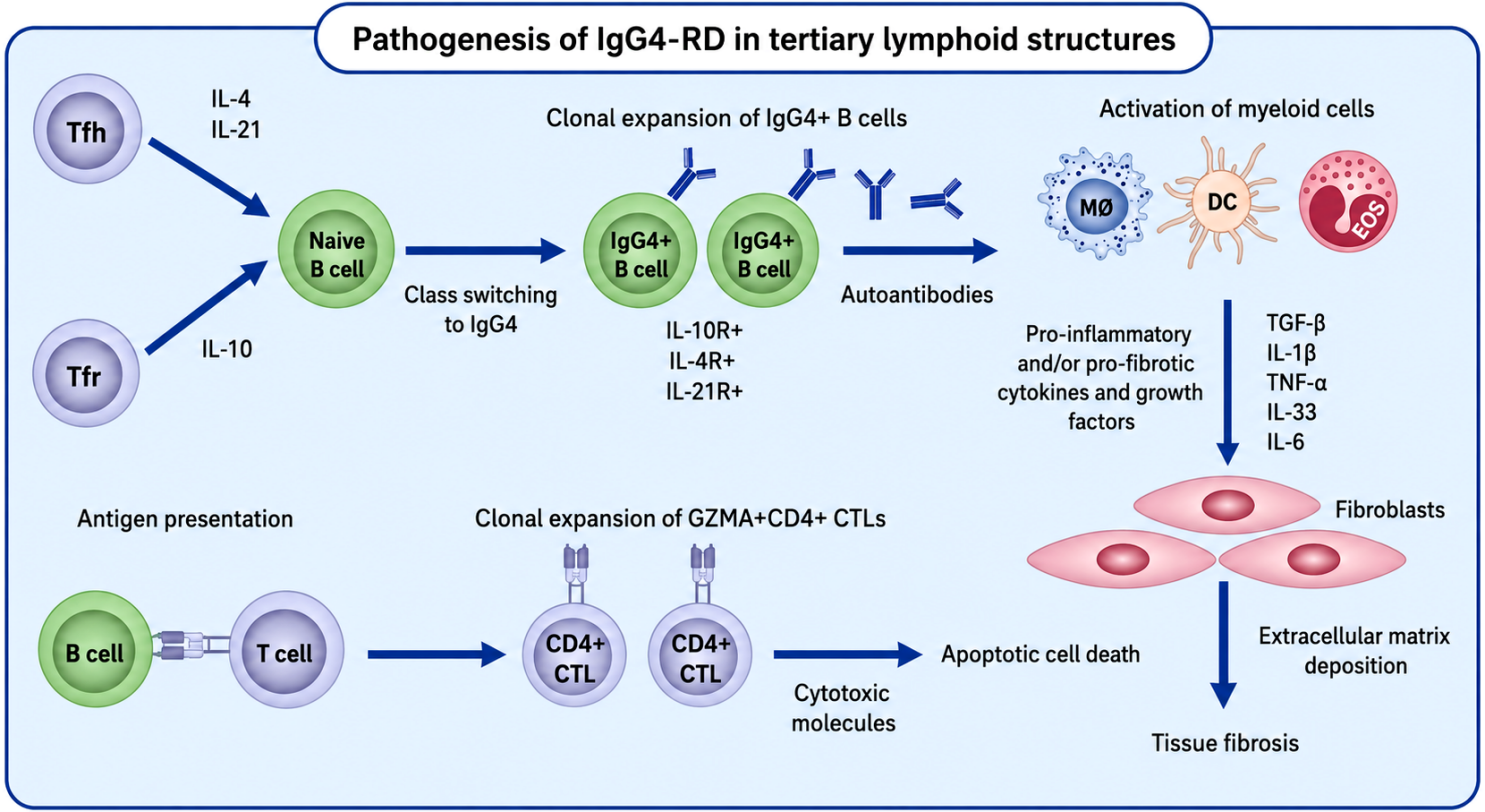
The mediators of innate and adaptive immunity involved in IgG4-related disease and allergic disorders (20).

The proposed pathogenic mechanisms underlying IgG4-related disease involve the coordinated action of cytokines, including IL-4, IL-21, and IL-10, produced by T follicular helper and T follicular regulatory cells, which drive immunoglobulin class switching of naive B cells toward IgG4-expressing memory B cells and plasmablasts. These activated B-cell subsets contribute to autoantibody production and antigen presentation, thereby promoting the clonal expansion of cytotoxic CD4^+^ T cells. In parallel, the activation of myeloid cell populations, including macrophages, eosinophils, and dendritic cells, leads to the release of pro-inflammatory and pro-fibrotic mediators. Collectively, these processes orchestrate tissue injury, extracellular matrix deposition, and progressive fibrosis characteristic of the disease (**Figure 1**) (20).

## Pathology

There are three major histopathological features associated with IgG4-RD: dense lymphoplasmacytic infiltration; fibrosis, arranged at least focally in a storiform pattern; and obliterative phlebitis. An increased number of eosinophils was also described (7).

Lymphoplasmacytic infiltration is characterized by small lymphocytes, predominantly T cells, with scattered aggregates of B cells. Plasma cells are also an essential component and eosinophils are found in mild to moderate quantities. Scattered macrophages may also be present (7).

Storiform fibrosis resembles the spokes of a cartwheel with spindle cells radiating from a center. The spindle cells (fibroblasts or myofibroblasts) are typically buried within the lymphoplasmacytic infiltration (7).

Obliterative phlebitis is characterized by a dense lymphoplasmacytic infiltration in the venous lumen and venous wall. Partially obliterated veins with transmural inflammatory infiltration are also consistent with the diagnosis of IgG4-RD. Occasionally, arteritis have also been described, characterized by a non-necrotizing lymphoplasmacytic infiltrate with or without obliteration of the arterial lumen (7).

## Pathophysiological correlations between IgG4-related disease, autoimmune pancreatitis, and allergic diseases

IgG4-RD is a systemic fibroinflammatory condition characterized by tumefactive lesions, dense lymphoplasmacytic infiltrates enriched in IgG4-positive plasma cells, and varying degrees of fibrosis (8). Type 1 autoimmune pancreatitis (AIP) is now widely recognized as the pancreatic manifestation of IgG4-RD, sharing its histopathological, serological, and immunological features (21). In contrast, type 2 AIP represents a distinct entity, lacking IgG4 elevation and systemic involvement, and is not considered part of the IgG4-RD spectrum (21).

A growing body of evidence supports the existence of shared immunopathogenic mechanisms between IgG4-RD, AIP, and allergic diseases, particularly involving a T helper 2 (Th2)-dominant immune response (22, 23). In IgG4-RD, antigen-driven immune activation promotes the expansion of Th2 cells, which secrete interleukins IL-4, IL-5, and IL-13. These cytokines play a central role in class-switch recombination of B cells to IgG4-producing plasma cells, as well as in the recruitment and activation of eosinophils, mast cells, and basophils—key effector cells also implicated in allergic inflammation (22, 23).

In parallel, regulatory T cells (Treg) contribute significantly to disease pathogenesis through the secretion of IL-10 and transforming growth factor-beta (TGF-β). These cytokines exert immunomodulatory effects, promoting IgG4 production while also facilitating tissue fibrosis, a hallmark of IgG4-RD (8, 23, 24). Additionally, T follicular helper (Tfh) cells, particularly the Tfh2 subset, are critically involved in germinal center reactions and B-cell differentiation, further amplifying IgG4 responses (23, 25).

The immunological overlap with allergic diseases is underscored by the frequent presence of atopy, elevated serum IgE levels, and peripheral eosinophilia in patients with IgG4-RD (22). Both conditions share a common Th2-skewed cytokine milieu, suggesting that chronic antigenic stimulation—potentially from environmental or autoantigens—may drive parallel or intersecting immune pathways. Moreover, innate immune mechanisms, including activation of dendritic cells and macrophages, as well as epithelial-derived cytokines such as IL-33 and thymic stromal lymphopoietin (TSLP), may further enhance Th2 polarization and sustain chronic inflammation (23, 26).

Clinically, these shared pathways have important implications. The overlap between IgG4-RD and allergic diseases may complicate diagnosis, as symptoms can be non-specific and multisystemic. However, recognizing this immunological continuum may aid in identifying biomarkers and tailoring therapeutic strategies. For instance, corticosteroids remain the first-line treatment for both IgG4-RD and AIP, while emerging biologic therapies targeting B cells or Th2 pathways (e.g., anti-CD20 or anti-IL-4/IL-13 agents) may offer promising alternatives in refractory cases (8, 23, 25).

### Clinical manifestation

Most people with IgG4-RD are asymptomatic, but IgG4-RD can affect nearly any organ, so there are many patterns of presentation. Four typical presentations are most common: hepatopancreatobiliary disease (31%), systemic disease (22%), head and neck disease (24%), and retroperitoneal disease (24%) (10, 27, 28). The main mediators of innate and adaptive immunity involved in IgG4-RD and allergic disorders are summarized in **Table 1**.

**Table 1:** Mediators of innate and adaptive immunity in IgG4-RD and allergic disorders (22).

Mediators of innate and adaptive immunity	Roles in IgG4-RD	Roles in Allergic disorders
Memory CD4+ T cells	Secretion of profibrotic cytokines, e.g., IL-6, IFN-γ and TGF-β, promoting induction of IgG4 class-switching, expansion of plasmablasts, and production of autoantibodies (4, 22, 25, 29). Secretion of IL-4, IL-5, IL-13 promoting eosinophil infiltration (30).	Secretion of pro-allergic cytokines, IL-4, IL-5, and IL-13, promoting both immunoglobulin E (IgE)– and eosinophil-mediated immune responses (8).
T follicular helper cells	Promotion of isotype class switching to IgE and IgG4, and ectopic GC formation through IL-21 production (22, 28).	Secretion of IL-4 and promotion of isotype switching to IgE (27).
B cells	Activation of B cells results in class switching from IgM to IgE and/or IgG4 (31).	Activation of B cells results in the production of IgG, IgA, and IgE antibodies in allergy (32).
Mast cells	Mast cell activation by high-affinity FcϵRI receptor crosslinking might contribute to fibrosis in IgG4-RD via degranulation and release of several granule-associated molecules, e.g., histamine, and TGF-β (33, 34).	Type I hypersensitivity allergic reactions are mediated by cross-linking of antigen-specific IgE immune complexes and FcϵRI receptors on the membrane surface of mast cells (106, 107).
Basophils	TLR2 and/or TLR4-activated basophils may promote IgG4 production via TLR signaling (35).	Basophils produce cytokines, such as IL-4 and IL-13, and release histamine and leukotriene after activation of FcϵRI by IgE crosslinking (36).
Eosinophils	Eosinophils may contribute to IgG4-RD pathogenesis by inducing fibrosis via the production of TGF-β and IL-13 (37).	Eosinophils produce IL-13 and TGF-β, which are involved in the pathogenesis of allergic diseases. Eosinophils also directly activate mast cells (145).
IgG1	Decreased complement levels in IgG4-RD may involve complement fixation by IgG1-containing immunocomplexes (38). Antagonistic function of IgG4 antibodies against pathogenic IgG1 autoantibodies noted when injected from a patient with autoimmune pancreatitis into neonatal mice and immunostaining in pancreatic tissue was performed (34).	Majority of IgE+ cells arise from somatically hypermutated IgG1-expressing cells, as demonstrated from analysis of Ig heavy regions in peripheral blood of patients with allergy (39). Binding of IgG1-containing immunocomplexes to the IgG inhibitory Fc gamma receptor (FcγRIIB) suppresses complement C5aR-mediated inflammatory signaling in allergy (33).
IgG4	Pathogenic role in IgG4-RD (1, 2).	Thought to promote tolerance in the context of food allergy (40).
IgE	IgE-positive mast cells might contribute to fibrosis in IgG4-RD, given their presence in IgG4-related fibrosclerotic mesenteric masses (5).	IgE sensitizes mast cells to release biologically active mediators such as histamine and prostaglandins in an antigen-specific manner in allergic diseases (41).
IFN-gamma	IFN-γ may contribute to chronic inflammation and fibrosis in IgG4-related dacryoadenitis and sialoadenitis (26).	Patients with severe asthma develop significantly reduced IFN-γ production in response to allergens that may result in increased IgE production by either switch of B cells into IgE-producing plasma cells or differentiation of CD4+ T-cells into Th2 cells (42).
Type 2 cytokines (IL-4, IL-5, IL-13)	Type 2 cytokines are up-regulated in the tissue of IgG4-RD, promoting peripheral blood eosinophilia and activating B cells to class switch from IgM to IgE and/or IgG4 (32, 43, 44).	Type 2 cytokines recruit effectors like mast cells, basophils, ILC-2 cells and eosinophils in allergy (12). Type 2 cytokines promote Ig isotype switch in B cells, resulting in production of allergen-specific IgE (7).
Alarmins (TSLP, IL-33)	Alarmins may contribute to chronic inflammation and IgG4+ B cell accumulation via induction of mucosal Type 2 immunity (10, 30, 45).	Alarmins drive allergic inflammation by triggering Type 2 cytokines (8–12).

IL-6, interleukin 6; IFN-γ, interferon gamma; TGF-β, tumor growth factor beta; IL-4, interleukin 4; IL-5, interleukin 5; IL-13, interleukin 13; GC, germinal center; IL-21, interleukin 21; IgE, immunoglobulin E; IgM, immunoglobulin M; IgG4, immunoglobulin G subtype 4; IgA, immunoglobulin A; TLR, toll-like receptor; FcγRIIB, Fc gamma receptor IIB; C5aR, complement 5a receptor; ILC-2, type 2 innate lymphoid cells; IgG4-RD, IgG4-related disease; TSLP, thymic stromal lymphopoietin; IL-33, interleukin 33; Th2, T helper 2 cells.

Type 1 AIP (lymphoplasmacytic sclerosing pancreatitis) is one of the most common manifestations of IgG4-RD, and 20% of these patients also have biliary manifestations (sclerosing cholangitis or biliary compression from the pancreas). The most common presentation is painless obstructive jaundice (in 70%), secondary to edema and pancreatic duct infiltration, along with weight loss, low-grade fever, fatigue, diarrhea, and exocrine or endocrine pancreatic insufficiency (10, 21, 46, 47). IgG4-related sclerosing cholangitis is the second most common disease within the IgG4-RD, sharing similar clinical and pathological features with AIP. The main clinical manifestations are abdominal pain, jaundice, and pruritus, but approximately 25% of patients may be asymptomatic (47). IgG4-related autoimmune hepatitis has also been described (48). This represents a subtype of autoimmune hepatitis but is characterized also by an aggregation of IgG4-expressing plasma cells in liver tissues (28). Type 1 AIP is frequently accompanied by an IgG4-related gastropathy, the gastric tissues presenting a prominent infiltration of IgG4-positive plasma cells (28).

A lot of people with IgG4-RD have multiple organ involvement. The most common sign is swelling of or within the organ, caused by an inflammatory pseudotumor. Lymph node involvement produces a generalized lymphadenopathy in 40%–80% of patients. Inflammatory pseudotumor can affect the lung. At the thyroid, it can present as Riedel thyroiditis or a fibrosing Hashimoto thyroiditis (28). The kidney can be affected by tubulo-interstitial nephritis, membranous glomerulonephritis, or an inflammatory pseudotumor. The last is characterized by pain due to kidney enlargement, with varying degrees of hematuria and proteinuria (49). It can also affect the skin (appearing erythematous plaques, papules, subcutaneous nodules, often itchy, usually on the face and forearms) and the prostate. In IgG4-RD, allergic manifestations like asthma can also appear. Many patients have atopic disease like seasonal allergies (10, 22, 50). However, the significance of atopic disease in IgG4-RD is poorly understood. Neoplasms have been reported to occur simultaneously or develop later in patients with IgG4-RD. Of those diagnosed at the same time with IgG4-RD, gastric, colorectal, and prostate neoplasms are those most commonly encountered, and of those discovered subsequently, lung cancer is the most frequently found, but a cause/effect relationship between IgG4-RD and cancer has not been found (9).

Head and neck disease manifests commonly as painless swelling of the salivary and lacrimal glands, lymphadenopathy, orbital myositis, and orbital pseudotumor, which often causes proptosis. Salivary and lacrimal involvement produces Mikulicz disease of the lacrimal glands (dacryoadenitis) or salivary glands (sialoadenitis). Mild dry mouth and dry eye symptoms may accompany glandular enlargement, making it easily confused with Sjögren’s syndrome (28). Less common manifestations include pachymeningitis or hypophysis. This form of IgG4-RD occurs more often in women (10).

Retroperitoneal or Ormond’s disease is characterized by fibrosis and less inflammation involving the aorta (periaortitis, inflammatory aortic aneurysm), the iliac arteries, and often the ureters, provoking hydronephrosis and even renal failure. Generally, patients are asymptomatic (10), but they can present vague pain in the back, flank, or lower abdomen, as well as lower limb edema (28).

### Diagnosis

The diagnosis of IgG4-RD is difficult because of the diversity of localization (29). The 2019 ACR/EULAR Classification Criteria are helpful (51). IgG4-RD should be suspected when a patient presents with a mass lesion or wall thickening in a characteristic organ (pancreas, salivary glands, bile ducts, orbits, kidney, lungs, aorta, retroperitoneum, pachymeninges, thyroid gland) (10, 47). Although nearly any organ can be affected, other localizations are unusual.

In most cases, the diagnosis is confirmed by biopsy (see above); unfortunately, this is not always possible because of the lesion localization or procedure risk. Characteristic pathologic features include a dense lymphoplasmacytic infiltrate rich in IgG4+ plasma cells and CD4^+^ T cells. This infiltrate is often accompanied by fibrosis that has a storiform pattern. (The word *storiform* is derived from the Latin word *storea*, or woven mat, describing the irregular, whorled pattern of fibrosis). Obliterative phlebitis with obstruction of the venous lumen with immune cell infiltration is also characteristic (7). In patients in whom affected organs are not amenable to biopsy, minor salivary gland (lip) biopsy should be considered. Even without clinical evidence of major salivary gland swelling or sicca symptoms, minor salivary gland biopsy can be a minimally invasive way to reach a histological diagnosis in some patients (52). IgG4 immunostaining is an essential test for the diagnosis of IgG4-RD, especially in cases without an elevated concentration of serum IgG4. It is a simple, highly reproducible test that provides strong confirmatory evidence for the diagnosis (6). The IgG4+/IgG+ plasma cell ratio >40% is a more powerful tool than IgG4+plasma cell counts in establishing the diagnosis of IgG4-RD. However, a >40% ratio is not enough for the diagnosis of IgG4-RD, because other diseases can also have such an elevated ratio, such as Castleman disease and rheumatoid arthritis (7, 53). Regardless of biopsy findings, the diagnosis is established by clinicopathological correlations (10).

Although a high level of serum IgG4 is not characteristic of IgG4-RD, a meta-analysis evidenced a sensitivity of 86% and a specificity of 90%, with IgG4 being the best single serum marker associated with the disease (54). Based on the studies of other authors, the sensitivity of serum IgG4 ranges between 50% and 90% and the specificity is 60% (9, 41, 55). High levels of serum IgG4 can also be found in some respiratory diseases (bronchiectasis, asthma), in sarcoidosis, chronic sinusitis, biliary diseases (primary sclerosing cholangitis, cholangiocarcinoma, lithiasis), in chronic pancreatitis with other etiologies, liver cirrhosis, autoimmune diseases (Sjögren’s syndrome, systemic lupus erythematosus, rheumatoid arthritis etc.) (9). Additional laboratory findings, such as eosinophilia, elevated IgE, polyclonal hypergammaglobulinemia, and hypocomplementemia, can also be useful (12, 40, 52), as well as flow cytometric detection of plasma blasts (52).

Classic radiography is often useful, as well as CT, MRI, and PET-CT (56). In type 1 AIP, there is diffuse “sausage-like” or segmental enlargement of the pancreas, often with a “halo”; in the kidneys, wedge-shaped hypodensities appear; ductal organs such as the bile duct or bronchus show diffuse “pipe-stem” wall thickening, as well as thickened aortic wall, hepatic mass lesions, and retroperitoneal fibrosis (52).

No single finding by examination, pathology, imaging, or laboratory tests is enough for diagnosis.

Patients with pancreatic localization of the disease, in addition to the ACR/EULAR Classification Criteria, can also be evaluated using the HISORT criteria (histology, imaging, serology, other organ involvement, response to therapy) (7, 10, 57). AIP presents with either diffuse or focal involvement. Diffuse involvement includes diffuse enlargement with loss of lobulations, long pancreatic duct strictures traversing more than one-third of the pancreas without downstream dilatation, and a hyperenhancing thin rim surrounding the pancreas, known as the halo sign. Focal involvement usually presents as a mass in the head of the pancreas, leading to common bile duct dilatation (7).

The updated comprehensive diagnostic criteria for IgG4-RD can be consulted (58).

### Differential diagnosis

Differential diagnosis is necessary with other diseases with increased numbers of IgG4+ plasma cells in tissue: inflammatory conditions (e.g., oral inflammatory diseases, primary sclerosing cholangitis, inflammatory bowel disease, rhinosinusitis, Rosai–Dorfman disease) (7, 52) and malignancies (lymphoma, myeloma, pancreatobiliary cancers). Also, peritumoral tissue rich in IgG4+ plasma cells can mimic IgG4-RD (7). An elevated serum IgG4 level can also be found in a number of respiratory diseases (bronchiectasis, tuberculosis, interstitial lung disease, asthma, chronic sinusitis), sarcoidosis, biliary diseases (primary sclerosing cholangitis, cholangiocarcinoma, lithiasis), autoimmune diseases (Sjögren’s syndrome, systemic lupus erythematosus, rheumatoid arthritis, inflammatory myopathies, and vasculitides), chronic pancreatitis of other etiologies, cirrhosis, allergic diseases (atopic dermatitis), and infections (9, 39, 52, 54, 59).

One of the main differential diagnoses is Sjögren’s syndrome: it begins with sicca symptoms and has a clearly defined serological profile of autoimmunity and typical histopathology (9, 39, 52).

Differential diagnosis is also necessary with multicentric Castleman disease, which also presents a high frequency of lymphadenopathy, tissue infiltration with IgG4 plasma cells, and high serum levels of IgG4, but the histological features are distinct (52).

Differential diagnosis of type 1 autoimmune pancreatitis (IgG4-RD) is necessary with type 2 autoimmune pancreatitis, characterized by neutrophilic infiltrates and occasionally epithelioid granulomas (7, 60), as well as with pancreatic cancer (9, 61).

Differential diagnosis is also necessary between IgG4-related sclerosing cholangitis, primary sclerosing cholangitis, and cholangiocarcinoma, with ERCP being the gold standard. IgG4-related sclerosing cholangitis typically presents with segmental stenosis of the lower common bile duct (28).

### Treatment and monitoring

The goal of treatment in IgG4-RD is to reduce disease activity, prevent irreversible damage, and induce and maintain remission. The response to treatment can vary based on the involved organ, the duration of disease, and the degree of fibrosis (10). First-line therapy includes glucocorticoids. The usual initial dose is 0.5–1 mg/kg/day of prednisone, based on the severity of the presentation. This initial dose is continued 2–4 weeks and then tapered off over 2–3 months (10, 33, 38). Based on clinical manifestations, radiological findings, and laboratory results, the dose is reduced by 5 mg every 1–2 weeks. The aim is to taper the dose to 5 mg/day over a period of 3–6 months (28). A short course of glucocorticoids for up to 2–3 months can be used for patients with severe or urgent disease (10, 19).

Because of the many secondary effects and potential toxicity of glucocorticoids (osteoporosis, diabetes mellitus, infections), conventional DMARDs (disease-modifying anti-rheumatic drugs) are often combined with glucocorticoids for induction therapy. Combination therapies with mycophenolate mofetil, cyclophosphamide, or leflunomide plus glucocorticoids have shown effects comparable to glucocorticoids alone (31, 34, 43). Other studies have also reported the benefits with methotrexate, azathioprine, hydroxychloroquine, tacrolimus, 6-mercaptopurine, and iguratimod (27, 30, 32, 44). The purpose of their use is to reduce the dosage of steroids and decrease the recurrence rate of the disease. Biological DMARDs such as rituximab, inebilizumab, or dupilumab have also been studied. The usual practice is to induce remission with rituximab. Very good results were obtained in a cohort study of type 1 AIP, in which 66.7% of patients entered full remission and 33.3% partial remission of the disease, while in a 17-month follow-up period, no patients relapsed (62). In another study, the relapse rate was 80% between 1 and 3 years of treatment with rituximab (63). Through various mechanisms, including antibody-dependent cell-mediated cytotoxicity, antibody-independent cell phagocytosis, and complement-independent cytotoxicity, rituximab orchestrates the elimination of B cells (28).

A new therapeutic possibility is the use of Bruton’s tyrosine kinase inhibitors like rilzabrutinib or zanubrutinib, which play a pivotal role in regulating B-cell proliferation and differentiation by thwarting signaling pathway transduction mediated by B-cell receptor activation. Also, bortezomib, a proteasome inhibitor, has showcased therapeutic success (28, 36).

While rituximab and Bruton’s tyrosine kinase inhibitors act on B cells, it is logical to try to influence different T cells that play an important role in the pathogenesis of IgG4-RD. These include helper T cells 2, regulatory T cells, and follicular helper T lymphocytes, all of which play crucial roles in disease progression. Abatacept is a recombinant fusion protein with its mechanism of action involving cytotoxic T lymphocyte-associated protein 4, inhibiting the activation of T cells and holding potential for the treatment of IgG4-RD. Elotuzumab selectively inhibits CD4^+^ cytotoxic T lymphocytes and has shown efficacy in the treatment of IgG4-related sclerosing mesenteritis (28, 64).

Patients with life-threatening manifestations, multi-organ disease, high values of serum IgG4, or elevated eosinophilia are at high risk for relapse and may benefit from maintenance therapy during remission (37, 65). The approach to maintain remission should be individualized based on the patient’s specific manifestations, history of damage, comorbidities, and other factors. Maintenance therapy may consist of low-dose glucocorticoids (<10 mg/day of prednisone) or rituximab 1 g every 6 months (10).

To this end, the therapy must be tailored to the affected organ: thyroxine in thyroiditis, pancreatic enzymes in pancreatic insufficiency, insulin in diabetes mellitus, and hydrocortisone, thyroxine, growth hormone, desmopressin, and sex hormones (testosterone or estrogen and progesterone) in hypopituitarism (10).

In some cases, such as orbital pseudotumor or sclerosing mesenteritis, surgical intervention may be necessary (38), including biliary drainage (28).

Close monitoring of disease activity is important to confirm successful induction of remission and to assess for disease relapse requiring retreatment (10). Laboratory tests used to monitor disease activity include high serum IgG4 (but IgG4 can remain elevated even in the absence of disease activity) and IgE concentrations, low complement C3 and C4 levels, and high peripheral eosinophil counts. High bilirubin and alkaline phosphatase levels can be used to assess disease activity in cholangitis (10). Many patients have disease manifestations that are more apparent on imaging, and in these cases, serial imaging is often necessary. Also, new manifestations of the disease can occur. Therefore, disease activity should be reassessed routinely every 3–6 months, even in the absence of symptoms, given the frequency of asymptomatic IgG4-RD (10).

### Complications

IgG4-RD can cause irreversible organ damage due to untreated disease, mass effect on neighboring structures, or iatrogenic injury. Examples of such damage include sicca syndrome after resection of a salivary gland, proptosis from a fibrotic orbital pseudotumor, anosmia due to sinonasal involvement, large vessel aneurysms or dissections, chronic ureteral obstruction, chronic kidney disease (including end-stage kidney disease), portal hypertension and liver cirrhosis, biliary obstruction, and others (10). There are also reported cases of malignancies.

Patients with IgG4-RD have an increased risk of clonal B-cell lymphoma and pancreatic malignancy (1, 35, 66, 67). The most frequently damaged organ is the pancreas. Approximately 60% of patients have exocrine or endocrine damage (35). The majority of diabetes cases present even before glucocorticoid therapy (68). The exocrine pancreatic insufficiency is characterized by weight loss. Approximately 10% of the patients with type 1 AIP may develop pancreatic calcification or chronic pancreatitis (45, 58). Patients with chronic pancreatitis are also at increased risk for osteopenia, osteoporosis, and major micronutrient deficiencies (45). There is also one case with post-bulbar duodenal ulcer as a complication of type 1 AIP (69).

### Prognosis

Prognosis is variable. IgG4-RD may spontaneously resolve or persist with remitting and relapsing symptomatology. The suggested risk factors of relapse are high serum IgG4 levels before treatment, persistently high serum IgG4 levels also after steroid treatment, and extensive multi-organ involvement (39). Fibrotic disease and chronic pancreatitis are often irreversible. Accurate diagnosis and staging are crucial because some patients have asymptomatic but potentially organ- or life-threatening diseases such as retroperitoneal fibrosis, peri-aortitis, or coronary arteritis, the last of which may develop suddenly (52). If untreated or undertreated, IgG4-RD can cause irreversible organ damage and even death (10).

### IgG4 in allergic reactions

IgG4 antibodies have an important role in the development of tolerance in atopic patients. It has been shown that beekeepers develop an IgG4-based response to bee venom associated with reduction of symptoms after exposure to the toxin, as well as in other cases of occupational exposure (1–3). Furthermore, allergen-specific immunotherapy is based on the development of IgG4 antibodies against allergens through regular and incremental exposure to allergenic antigens (1, 70). IgG4 competitively binds to allergens, thus hindering the formation of IgE–antigen immune complexes (1, 71, 72). Furthermore, it also prevents the degranulation of mast cells and the cascade that would lead to allergic responses (73, 74). It was demonstrated that IgG4 antibodies commonly arise after long-term exposure to an antigen by a modified Th2 response such as in a scenario of allergen desensitization therapy. Thus, the production of IgG4 antibodies can reduce the degree of chronic allergic or inflammatory reactions to environmental stimuli by displacing the binding of IgG1 or IgE antibodies with their cognate antigens or allergens (75, 76). It is well established that there are strong links between IgG4 and IgE. Usually, IgG4 responses are connected with IgE-mediated allergic reactions since both antibodies are induced by Th2 cytokines, mainly IL-4 and IL-13 (76, 77). However, the two cytokine receptors are distinct since the production of IgE antibodies often occurs before IgG4 (78). Thus, a modified Th2 response is defined as the “presence of IgG4 antibodies in the absence of IgE antibodies.” This antiallergic effect is produced by attenuation of Th2-cytokine-mediated inflammation and immunosuppression (76, 79). It was reported that DNA encoding IgG4 is upstream to the DNA encoding IgE and is deleted during the class switch to IgE in normal situations (80). It was also demonstrated that the key cytokine driving “reverse” IgE/IgG4 class switch in the modified Th2 response is IL-10, which promotes IgG4 production but inhibits IgE production (76, 81). Other investigations have demonstrated that, in the context of an allergic response, IgE-producing plasma cells require not only IL-4 but also IL-5, IL-6, IL-7, IL-9, and IL-13 to help synthesize IgE (81). In addition to Th2 cytokines, the T follicular helper 2 (Tfh2) cell-derived cytokine, IL-21, is also involved in determining the IgG4/IgE ratio (76, 82, 83). It was also confirmed that the IgG4/IgG ratio was statistically higher in the presence of IL-4 alone than in the presence of Tfh2 cytokines alone, including IL-21, in patients with IgG4-RD, but not in healthy individuals. Also, IL-10 is implicated in the class switch from IgE to IgG4 production.

Furthermore, IL-10 is needed for driving the differentiation of IgG4-class-switched B cells to IgG4-secreting plasma cells. IL-21 also exerts a similar effect to IL-10 and is one of the major cytokines that drive the IgG4 shift (76, 84). It was described that human IgG4 antibodies against allergens are unable to link across identical antigens, in which functional change to monovalency of IgG4 antibodies is suspected. This unique immunological property can be applied to allergen-induced immunotherapy and the production of IgG4 therapeutic antibodies in clinical practice (76, 85–87).

IgG4 antibodies are also involved in the maintenance of eosinophilic esophagitis and eosinophilic chronic rhinosinusitis. Histological levels of IgG4 were found to be significantly lower during remission when compared to samples collected during disease activity (88, 89).

It is well-known that most of the antibody-mediated autoimmune diseases are caused by IgG1 and IgG3, but in some autoimmune diseases, like pemphigus vulgaris, idiopathic membranous nephropathy, or myasthenia gravis, IgG4 is involved in the pathogenesis of these diseases (90–93).

## Conclusions

IgG4-RD is a relatively recently described condition whose etiology and pathogenesis are unclear. It is characterized by lymphoplasmacytic infiltrate with predominant IgG4+ cells and fibrosis in different organs. Most frequently involved are the pancreas, lymph nodes, and salivary glands, but almost all of the organs can be affected. The majority of patients have elevated serum IgG4 levels, but despite the name, the role of IgG4 in this disorder remains unclear. The treatment consists of systemic corticosteroids, and biological therapy with rituximab is effective; however, newer strategies may include blockade of the modified Th2 response and novel anti-fibrogenic therapies (77). Despite recent developments in IgG4-RD research, essential questions remain unanswered (1).
